# Genome-Wide Association Study of Body Weight Traits in Inner Mongolia Cashmere Goats

**DOI:** 10.3389/fvets.2021.752746

**Published:** 2021-12-01

**Authors:** Lei Zhang, Fenghong Wang, Gong Gao, Xiaochun Yan, Hongfu Liu, Zhihong Liu, Zhixin Wang, Libing He, Qi Lv, Zhiying Wang, Ruijun Wang, Yanjun Zhang, Jinquan Li, Rui Su

**Affiliations:** ^1^College of Animal Science, Inner Mongolia Agricultural University, Hohhot, China; ^2^Inner Mongolia Jinlai Livestock Technology Co., Ltd, Hohhot, China; ^3^Key Laboratory of Animal Genetics, Breeding and Reproduction, Hohhot, China; ^4^Key Laboratory of Mutton Sheep Genetics and Breeding, Ministry of Agriculture and Rural Affairs, Hohhot, China; ^5^Engineering Research Center for Goat Genetics and Breeding, Hohhot, China

**Keywords:** Inner Mongolia cashmere goats, genome-wide association study, GGP_Goat_70K SNP chip, birth weight (BW), weaning weight (WW), yearling weight (YW)

## Abstract

**Objective:** Body weight is an important economic trait for a goat, which greatly affects animal growth and survival. The purpose of this study was to identify genes associated with birth weight (BW), weaning weight (WW), and yearling weight (YW).

**Materials and Methods:** In this study, a genome-wide association study (GWAS) of BW, WW, and YW was determined using the GGP_Goat_70K single-nucleotide polymorphism (SNP) chip in 1,920 Inner Mongolia cashmere goats.

**Results:** We discovered that 21 SNPs were significantly associated with BW on the genome-wide levels. These SNPs were located in 10 genes, e.g., Mitogen-Activated Protein Kinase 3 (*MAPK3*), LIM domain binding 2 (*LDB2*), and low-density lipoprotein receptor-related protein 1B (*LRP1B*), which may be related to muscle growth and development in Inner Mongolia Cashmere goats. Gene Ontology (GO) and Kyoto Encyclopedia of Genes and Genomes (KEGG) enrichment analysis revealed that these genes were significantly enriched in the regulation of actin cytoskeleton and phospholipase D signaling pathway etc.

**Conclusion:** In summary, this study will improve the marker-assisted breeding of Inner Mongolia cashmere goats and the molecular mechanisms of important economic traits.

## Introduction

Body weight is one of the most important economic traits for livestock that can be measured during the entire animal lifetime from birth to slaughter. The early growth rate of a goat has a strong implication on both reproductive and production performances. Early growth performance traits, such as birth weight (BW) and weaning weight (WW), are the basis for selection in genetic improvement programs for meat production (1). To investigate the relationships between body weight and meat quality traits, PCR-restriction fragment length polymorphism and PCR-single strand conformation polymorphism were applied to suggest a low negative relationship between BW, meat quality, and genetic markers (*IGF-II* and *CAPN1*) in chickens breeding for meat quality (2). However, conventional breeding methods cannot make significant progress in a short period of time. To a large extent, it hinders the rapid industry development of animal production.

Genome-wide association study (GWAS) is a tool of genome-wide linkage disequilibrium to determine genetic marker information (e.g., single-nucleotide polymorphism, SNP, loci, and copy number) and related genes that genetically affect complex phenotypic traits or important quantitative economic traits throughout the genome (3–6). In 2005, the first GWAS report of macular degeneration of the retina was published in Science (7). With the completion of multi-species genome sequencing, the continuous development of high-throughput SNP chips, and genome typing technologies, GWAS has become a popular solution to the genetic positioning of complex human diseases and animal quantitative traits. For example, great progress has been made in GWAS research on obesity (8), coronary heart disease (9), diabetes (10), and other diseases, successfully identifying complex diseases significant SNPs. GWASs apply SNP arrays to accurately screen and identify the major genes of important economic traits, which becomes a prerequisite for rapid improvement for the molecular breeding of domestic animals, e.g., cattle, pigs, chickens, and sheep. For instance, A GWAS of five meat quality traits in 231 Yorkshire pigs identified 344 significant SNPs associated with five meat quality traits using PorcineSNP60 BeadChip, in which 323 SNPs were located in the reported QTL regions and 21 were novel (11). In a GWAS of the 600 K Affymetrix Axiom Chicken Genotyping Array, *KCTD4*, LIM domain binding 2 (*LDB2*)*, HEP21*, and *PCASP2* were closely correlated with the spleen weight of Layer chicken (12). Ghasemi et al. carried out a GWAS on the BW of Lori-Bakhtiari sheep and identified three genes (*RAB6B, Tf serotransferrin*, and *GIGYF2*) as candidate genes for this trait (13). GWAS of body weight in two cattle populations from the Russian Federation (Siberian region) was determined using the GGP HD150 and identified five statistically significant SNPs and the *CCND2* genes as the candidate gene for body weight trait (14).

The Inner Mongolia cashmere goat is a local breed in China with dual-purpose, producing both pleasant meat and excellent cashmere. It is famous for its “white cashmere and meat” in Inner Mongolia. Besides, Inner Mongolia cashmere goats also provide sufficient meat resources and have become one of the most important livestock species for the herdsmen. However, there are no GWAS studies focusing on the body weight traits of Inner Mongolia cashmere goats. Therefore, in this study, we examined BW, WW, and yearling weight (YW) by GGP_Goat_70K SNP chip and GWAS methodology for Inner Mongolia cashmere goat population of 1,920 individuals to identify significant SNPs and the major candidate genes associated with body weight traits in cashmere goats. This study may facilitate the potential use of major genes involving in growth and production traits for genetic improvement of productivity in cashmere goats.

## Materials and Methods

### Ethics Statement

In this experiment, the breeding environment was in compliance with the standards relevant to an ordinary animal laboratory facility in China National Standard “Laboratory Animal Environment and Facilities” (GB14925-2010). The feeding and the experimental operations on animals were in accordance with the animal welfare requirements.

### Phenotypic Measurements and Sample Collection

Goats used in the present study (*n* = 1,920, 2 years old from six herds; 1 year old from three herds) were selected randomly from the Arbas Stock Farm in Inner Mongolia, China. The BW, WW (3.5 months), and YW (12 months) were measured using an electronic scale for all goats. BW was recorded within 0.5 h after birth, while WW and YW were measured after 12 h of fasting. Samples of ear tissue were collected by ear deficiency forceps and quickly placed in a prepared cryopreservation tube containing 75% alcohol for storage at −80°C until DNA extraction.

### Statistical Analysis

Generally, the fixed effect of each trait in the model was identified by General Linear Model (GLM) procedure using the Statistical Analysis System (SAS) program. The influencing factors included the year of production (three levels, 2018-2020), herd (six herds, 1–6), birth status (simple or twin), maternal ages (six levels, 3–8), and sex (male and female, 1–2). Therefore, repeatability and multivariate animal model was used with restricted maximum likelihood method (AIREML) in the WOMBAT software to estimate the variance components of each trait. Then heritability for each trait was obtained under this model. The default convergence criterion was 10^−8^. The formula for this model was as follow:


$$\begin{array}{l} y_{i}=X_{i}b_{i}+Z_{i}a_{i}+Y_{i}m_{i}+W_{i}p_{i}+e_{i} \end{array}$$

Where *y*_*i*_ is the vector of observation of animals for trait *i*; *b*_*i*_ is the vector of fixed effects for trait *i*; *a*_*i*_ is the vector of direct additive genetic effects of animal for trait *i*; *m*_*i*_ is the vector of random maternal genetic effects of animal for trait *i*; and *p*_*i*_ is the vector of individual performant environmental effects for trait *i*. *Y*_*i*_ is maternal genetic effects for trait *i*, *X*_*i*_, *Z*_*i*_, and *W*_*i*_ are design matrices of the corresponding effects, respectively. *e*_*i*_ is the vector of random residuals effects for trait *i*. The heritability (h^2^), was calculated using the formula $h^{2}=\;\frac{\sigma_{a}^{2}}{\sigma_{p}^{2}}$.

### Genotyping and Quality Control

Deoxyribonucleic acid was extracted from ear tissue with the standard phenol-chloroform method according to the protocol of the manufacturer. DNA integrity and purity were tested using 2% agarose gel electrophoresis and a NanoDrop 2000 ultraviolet spectrophotometer (Thermo, Waltham, MA, USA).

The samples were genotyped using the Illumina Goat SNP 70K BeadChip panel that included 67,088 SNPs (Inner Mongolia Agricultural University, China). The samples with call rates <90% were removed from the analysis. The SNPs with GenCall (GC) scores <0.6, genotype call rates <90%, minor allele frequencies (MAF) < 0.01, and significant Hardy-Weinberg (HWE) disequilibrium at 10^−5^ were removed from the analysis. Plink 1.90 beta (15) and R software were used for quality control.

### GWAS

The correlations between the SNPs and the traits were tested using mixed linear models in GEMMA software version 0.98 (16–18). Therefore, herd and age were combined as fixed effects, as they all significantly related to body weight traits. The statistical analysis model used in this study was y = Xα + Zβ + Wμ + e, where y is the phenotypic trait, X is a matrix of fixed effects, α is the estimation parameter of the fixed effects, Z is a matrix of SNPs, β is the effect of the SNPs, W is a matrix of random effects, μ is the predicted random individuals, and e is the random error, with the distribution e~ N (0, $\delta_{\text{e}}^{2}$). The significance threshold for the GWAS was defined using the Bonferroni correction method. The suggestive genome-wide association significance threshold was *P* < 9.92 × 10^−7^ (0.05/50383), and the chromosome-wide significance level threshold was *P* < 2.88 × 10^−5^ (0.05/50,383/29). Chromosome-wide significance level SNPs were defined to call chromosome-wide significance associations, with a suggestive association corresponds cutoff *P* < 10^−4^ (19). The quantile–quantile (Q–Q) plots were visualized by plotting the distribution of obtained vs. expected log_10_ (*P*-value) with inflation factors (λ). The association map and the significant SNPs were visualized in the Manhattan plot with a threshold line. The Manhattan and Q–Q graphics were generated with R v. 3.5.2.

### Bioinformatics Analysis

Genes associated with significantly correlated SNP loci were annotated with the goat reference genome (ARS1, GCF_001704415.1). The genes were used as input for Cytoscape that analyzes and visualizes Gene Ontology (GO) and Kyoto Encyclopedia of Genes and Genomes (KEGG) (20, 21). GO terms and KEGG pathways with adjusting *P* < 0.05 were statistically significant.

## Results

### Descriptive Statistics and Heritability for Weight Traits of Cashmere Goats

Observed phenotypes were analyzed by first descriptive statistics (Table 1). The BW, WW, and YW ranged from 1.7 to 4.2, 16.5 to 29.5, and 24.0 to 49.5 kg, respectively. The coefficients of variation (CV) for BW, WW, and YW were 17.45, 57.93, and 37.98%, respectively. The results indicate that substantial phenotypic variation of these three traits exists in the population of Inner Mongolia cashmere goats. The heritability estimates for BW, WW, and YW were 0.10, 0.27, and 0.10, respectively.

**Table 1:** Descriptive statistics and variance components of body weight traits in Inner Mongolia cashmere goats.

**Traits**	***N***	**Mean**	**SD**	**Min**	**Max**	**CV (%)**	** $\sigma_{\text{a}}^{2}$ **	** $\sigma_{\text{c}}^{2}$ **	** $\sigma_{\text{m}}^{2}$ **	** $\sigma_{\text{e}}^{2}$ **	** $\sigma_{\text{p}}^{2}$ **	** ${\text{h}}_{\text{T}}^{2}\pm \text{S}\text{E}$ **
BW (kg)	1,917	2.75	0.48	1.70	4.20	17.45	0.02	0.01	0.02	0.14	0.19	0.11 ± 0.01
WW (kg)	1,868	17.52	10.15	16.50	29.50	57.93	1.57	0.91	0.88	2.51	5.87	0.27 ± 0.02
YW (kg)	1,892	36.10	13.71	24.00	49.50	37.98	0.19	0.14	-	1.55	1.88	0.10 ± 0.03

*$\sigma_{a}^{2}$, direct additive genetic variance; $\alpha_{c}^{2}$, individual permanent environment variance, $\sigma_{m}^{2}$, maternal genetic variance; $\sigma_{e}^{2}$, residual variance; $\sigma_{p}^{2}$, phenotypic variance; $h_{T}^{2}$, direct heritability; SE, standard error; BW, birth weight; WW, weaning weight; YW, yearling weight; CV, coefficients of variation*.

### Quality Control of Genotyping

The body weight traits of the 1,920 Inner Mongolia cashmere goats measured in this study included BW, WW, and YW. A total of 67,088 SNPs were genotyped. Subsequently, 2,655 SNPs were excluded from our dataset as they did not pass the HWE, while 10,446 SNPs were excluded from our dataset as they did not pass the HWE MAF tests (–MAF 0.01–HWE 1e^−5^). After the quality control (QC) was performed on the raw genotypes, a total of 1,909 animals and 50,383 SNPs were obtained and were distributed over the 29 goat chromosomes (Supplementary Figure 1). The descriptive statistics of the studied traits are listed in Table 1. All traits are normally distributed. The PCA result demonstrated that there were no genetic differences between the samples (Supplementary Figure 2).

### GWAS

**Figure 1:**
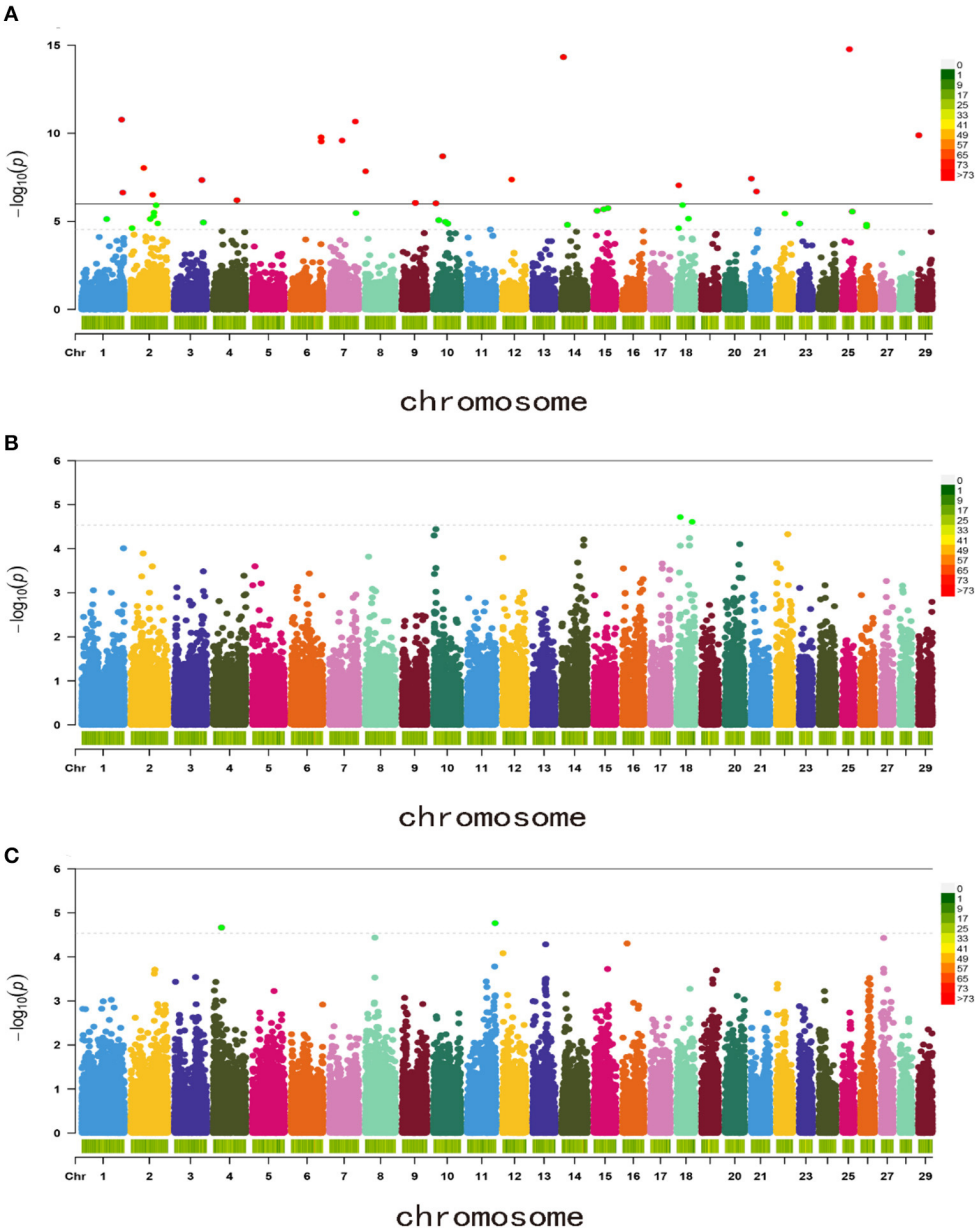
Manhattan plots of body weight traits for Inner Mongolia cashmere goats. **(A)** Birth weight (BW); **(B)** weaning weight (WW); and **(C)** yearling weight (YW).

Based on the 50,383 SNPs, GWAS was then performed with a mixed linear model for the BW, WW, and YW. A total of 21 SNPs reached the genome-wide significance levels for BW traits and 50 SNPs reached the chromosome-wide significance levels for the three traits (Figure 1). For BW, 21 genome-wide significance levels correlated SNPs were detected on the chromosomes Chr1, Chr2, Chr3, Chr4, Chr6, Chr7, Chr8, Chr9, Chr10, Chr12, Chr14, Chr18, Chr21, Chr25, and Chr29, these SNPs were electively annotated to 10 genes, e.g., Mitogen-Activated Protein Kinase 3 (*MAPK3*), *LDB2*,low-density lipoprotein receptor-related protein 1B (*LRP1B*; Figure 1A, Table 2).

**Table 2:** Significant single-nucleotide polymorphisms (SNPs), associated traits, and candidate genes identified in genome-wide association studies (GWASs).

**Trait**	**SNP ID**	**Chr**	**Position (bp)**	***P*-Value**	**Gene**
BW	26,093,025	25	26,093,025	1.69E-15	*MAPK3*
	snp2971	14	6,287,060	4.72E-15	–
	snp36371	1	1,476,313,984	1.69E-11	–
	96,351,932	7	96,351,932	2.15E-11	*ADGRE2*
	snp11977	6	112,102,568	2.92E-10	*LDB2*
	snp6911	2	81,102,675	3.10E-07	*LRP1B*
WW	snp41865	18	12,415,004	1.92E-05	*CRISPLD2*
	snp18295	18	56,522,928	2.45E-05	*TULP2*
YW	102,722,042	11	102,722,042	1.71E-05	*RAEP*
	snp20004	4	30,271,648	2.16E-05	–

*BW, birth weight; WW, weaning weight; YW, yearling weight*.

**Figure 2:**
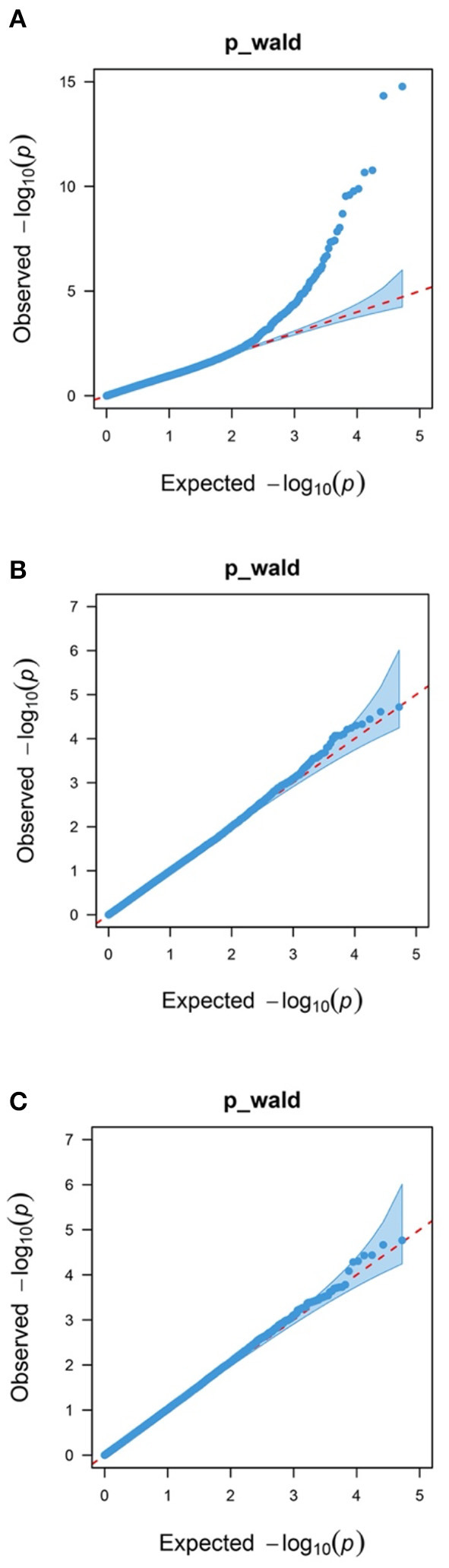
Quantile–quantile plots of body weight traits using a mixed linear model approach. Blue dots represent the –log_10_ (*P*-value) of the entire study, and the red line represents the expected values under the null hypothesis of no association. **(A)** Birth weight (BW); **(B)** weaning weight (WW); and **(C)** yearling weight (YW).

In addition, 46 correlated SNPs were identified at chromosome-wide significance levels. For WW, two chromosome-wide significance levels correlated SNPs identified on Chr18 were related to two genes, *CRISPLD2* and TUB-like protein 2 (*TULP2*, Figure 1B, Table 2). For YW, two chromosome-wide significance levels correlated SNPs were detected on Chr4 and Chr11, corresponding to one gene, progestogen-associated endometrial protein (*PAEP*; Figure 1C, Table 2). The Q–Q plot showed that the genomic inflation factors (λ) of BW, WW, and YW were 0.983, 0.990, 1.017, respectively (Figure 2), indicating no genome expansion. Based on the observed and expected *P*-values in the Q–Q plots of the three body weight traits, there were no population stratification phenomena. However, this phenomenon was significant for the SNPs, which were highly correlated with the three body weight traits.

### Functional Enrichment Analysis of GO and KEGG

**Figure 3:**
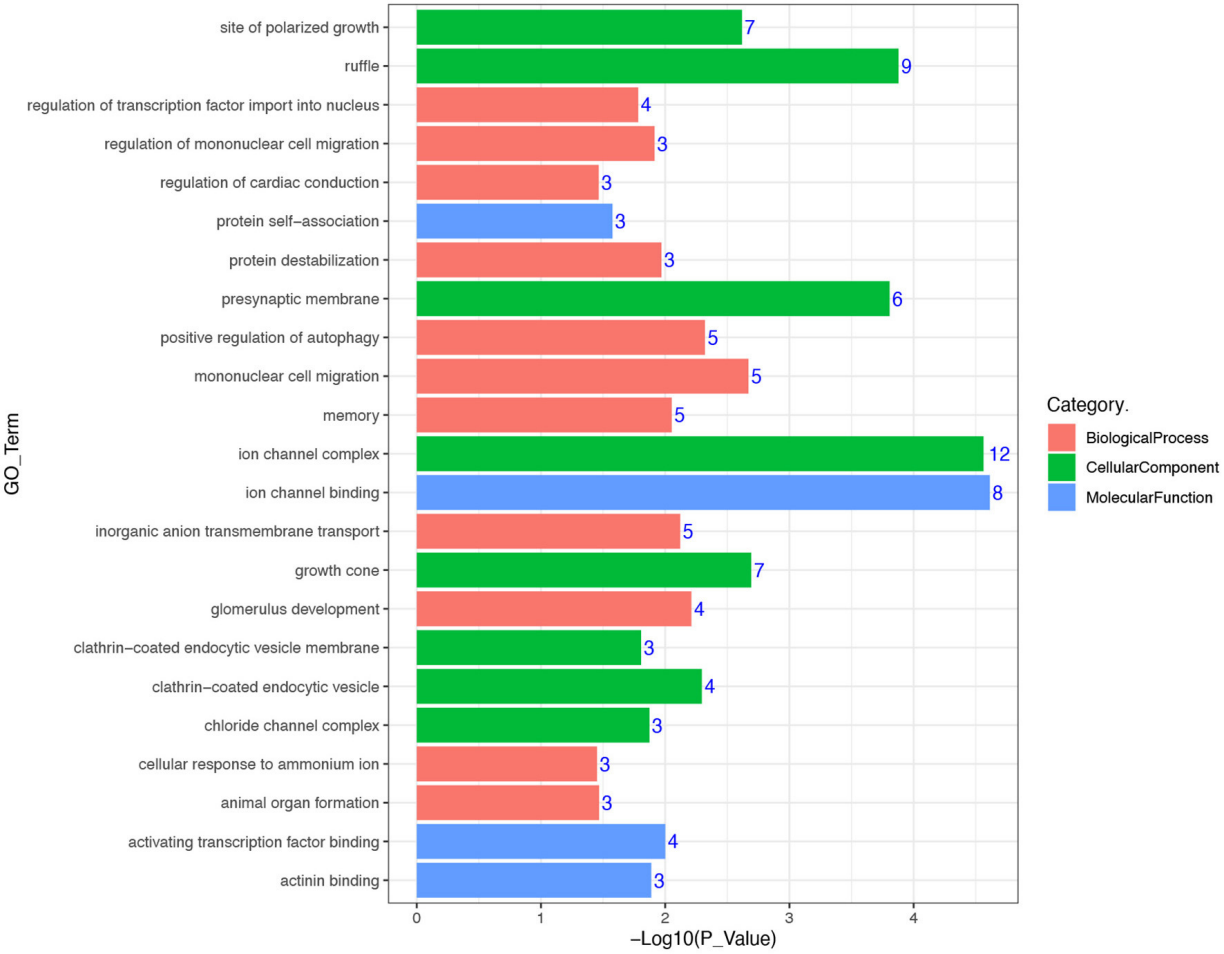
Gene ontology (GO) enrichment analysis for the regional candidate genes with chromosome-wide significant association.

**Figure 4:**
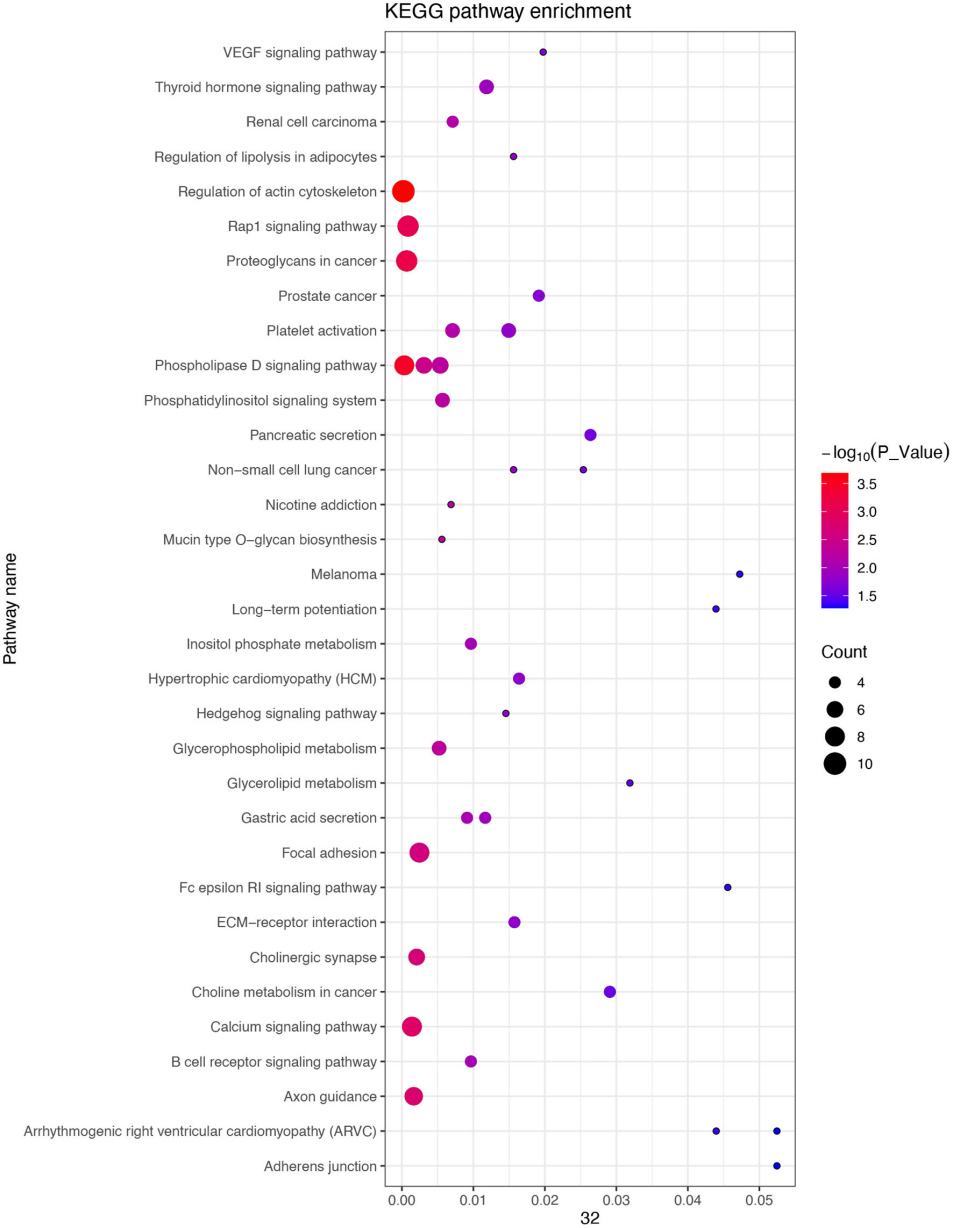
Kyoto Encyclopedia of Genes and Genomes (KEGG) enrichment analysis for the regional candidate genes with the chromosome-wide significant association.

In an attempt to better understand the biological functions and signaling pathways of the trait-associated genes, we performed GO and KEGG enrichment analysis for 13 genes near the SNPs of the three body weight traits, and uncovered enriched 23 GO terms, i.e., 11 biological processes, 8 cellular components, and 4 molecular functions (Figures 3, 4), which are closely correlated to growth and organ formation.

## Discussion

Body weight is an important economic trait in goat production. Therefore, it is important to understand the underlying molecular mechanisms behind goat body weight and identify important functional genes that influence cashmere goat production and breeding. However, compared with reported GWAS results for sheep body weight (6, 13, 22), the goat candidate genes of body weight are not well-studied. Therefore, in this study, genome-wide associations of BW, WW, and YW were determined using the GGP_Goat_70K SNP chip in 1920 Inner Mongolia cashmere goats.

In this study, the heritability of WW is moderately high (0.27), which is similar to the values estimated by Ma et al. (23). The heritabilities of BW and YW (0.11, and 0.10, respectively) are lower than the values estimated by Hossein and Mohammad et al. (24) (0.22 and 0.25, respectively), and Ma et al. (23) (0.647 and 0.571, respectively). The differences between studies are probably caused by the source of goat breeds, model design, and data structure.

Maternal age only has significant effects on BW and WW in early traits, but not YW, indicating great influences of maternal effects on lamb BW and WW. Studies discovered that with the increase of the age of mother, BW and WW also increase, suggesting lower BW and WW of lambs born to primiparous ewes than that of adult ewes. The reason is that the physical maturity and physical maturity of primiparous ewes are not well-developed. In short, lactating lambs have a strong dependence on the ewe, strengthen the feeding and management, improve the nutritional status, and promote lactation, which is conducive to the normal development of lamb.

To correct the effects of population structure and individual kinship relationships, a population genetic structure is considered as a fixed effect while an individual kinship is thought of as a random effect. Therefore, the GWAS of BW, WW, and YW of 1,920 Inner Mongolia cashmere goats was performed using a mixed linear model in this study. The Inner Mongolia cashmere goats that we chose are mainly grazing animals and the nutritional level of their pastures varies among the seasons, which will further affect gene expressions (25, 26).

Body weight growth is closely related to the growth of obesity, fat, and muscle. The candidate genes identified in this study were also closely correlated with growth and tissue development. For instance, the *TULP2* gene is located on chromosome 18 and is correlated with WW. *TULP2* is a member of the tubby gene family, and gene SNPs in the 19q13.33–13.43 chromosomal region are significantly related to severe obesity in French Caucasians (27). Jackson et al. demonstrate that *CRISPLD2* is a circulating adipokine that may regulate adipocyte remodeling during weight loss (28).

The *LDB2* gene is located on chromosome 6 and correlated with BW. *LDB2*, also known as CLIM-1, was identified as a LIM domain-associated cofactor and functions as a transcriptional regulatory factor (29–31). The LIM domain-binding factor 2 (*LDB2*) gene in the chicken chromosome 4 (GGA4) region ~8.6 Mb in length (71.6–80.2 Mb) had the strongest association with body weight for weeks 7–12 and with average daily gain for weeks 6–12 (32). Liu et al. identify *LDB2* genes on chicken (*Gallus gallus*) chromosome 4 associated with Carcass Weight (CW) and Eviscerated Weight (EW) traits, GWASs were conducted using the Illumina 60 K SNP Bead chip to genotype 724 Beijing-You chickens (33). In Wang's et al. they performed a new GWAS using specific locus amplified fragment sequencing (SLAF-seq) technology to discover that the *LDB2* gene in this region had a very strong association with body weight (34). Wei et al. indicated that a 31-bp indel located in the second intron region of the *LDB2* gene was significantly correlated with some growth traits and carcass traits of chickens (35).

The *LRP1B* gene is located on chromosome 2 and correlated with BW. Houde et al. found epigenetic variations at *LRP1B*, a gene associated with the development of obesity or cardiometabolic complications, are involved in fetal metabolic programming (36). Wang et al. performed a GWAS in 82 sows with an extreme SD of BWs within the first parity to identify *LRP1B* may contribute to the BW variability trait (37). *LRP1B* gene encodes *LRP1B* and mediates cellular cholesterol uptake (38). Dietrich et al. reported that knockout of *LRP1B* in mice results in early embryonic lethality (39). Association analysis identified *LRP1B* as a determinant of rat cholesterol concentrations in low-density lipoproteins (LDL) and a significant association with child body mass index (BMI) in humans (40). C*NR1* is involved in the growth cone (GO: 0030426). *CNR1* can affect the regulation of leptin signaling to regulate BW, food intake, and metabolism (41). Besides, the genes associated with SNP are involved in the biological process. *MAPK3* is involved in fundamental cellular processes, such as membrane trafficking, actin cytoskeleton remodeling, cell proliferation and cell survival, animal organ formation, and glucose metabolism. In this study, Prominin 1 (*PROM1*), *FBXL3*, and *LOC102176015* genes are new candidate genes related to BW. *PROM1* is a protein-coding gene, which plays a role in cell differentiation, proliferation, and apoptosis. *FBXL3* encodes a member of the F-box protein family, which plays a key role in the maintenance of both the speed and the robustness of the circadian clock oscillation.

## Conclusion

In summary, the first GWAS of Inner Mongolia cashmere goats was performed to identify gene-associated to three body traits. We identified 21 SNPs that reached the genome-wide significance levels and 50 SNPs that reached the chromosome-wide significance levels for the three traits in a population of 1,920 Inner Mongolia cashmere goats. *MAPK3, LDB2*, and *LRP1B* genes are key candidate genes for BW. *PROM1, FBXL3*, and *LOC102176015* genes are new genes related to BW and can be given to follow-up priority research. The candidate genes in this study were closely correlated to BW development. These findings will make a significant contribution to the understanding of the mechanisms underlying BW traits and facilitate genetics improvement of productivity in Inner Mongolia cashmere goats by providing essential genes related to goat BW traits.

## Data Availability Statement

The original contributions presented in the study are publicly available. This data can be found here: figshare.com/s/7734276ce6c1239e9474.

## Ethics Statement

All samples were collected in accordance with the International Guiding Principles for Biomedical Research involving animals and approved by the Special Committee on Scientific Research and Academic Ethics of Inner Mongolia Agricultural University, responsible for the approval of Biomedical Research Ethics of Inner Mongolia Agricultural University [Approval No. (2020) 056]. No specific permissions were required for these activities, and no endangered or protected species were involved.

## Author Contributions

All authors listed have made a substantial, direct, and intellectual contribution to the work and approved it for publication.

## Funding

This work was financially supported by National Natural Science Foundation of China (31860637); Science and Technology Major Project of Inner Mongolia Autonomous Region 2021SZD0010); Science and Technology Project of Inner Mongolia Autonomous Region (2019GG243); China Agriculture Research System of MOF and MARA (CARS-39); Scientific Project of Inner Mongolia Agricultural University on High-level Introduced Talented Personnel (NDYB2018-1); Central Government Guides Local Science and Technology Development Fund Projects (2020ZY0007); Youth Foundation in Inner Mongolia Agricultural University (No. QN202003). Special Funding Project for the Iconic Achievements of the College of Animal Science, Inner Mongolia Agricultural University (No. BZCG202111).

## Conflict of Interest

LZ and LH were employed by company Inner Mongolia Jinlai Livestock Technology. The remaining authors declare that the research was conducted in the absence of any commercial or financial relationships that could be construed as a potential conflict of interest.

## Publisher's Note

All claims expressed in this article are solely those of the authors and do not necessarily represent those of their affiliated organizations, or those of the publisher, the editors and the reviewers. Any product that may be evaluated in this article, or claim that may be made by its manufacturer, is not guaranteed or endorsed by the publisher.
