# Supplementary material for: Genome-Wide Association Study of Body Weight Traits in Inner Mongolia Cashmere Goats
Source: Front Vet Sci. 2021 Dec 1;8:752746. doi: 10.3389/fvets.2021.752746 (PMC8673091; doi:10.3389/fvets.2021.752746)
Supplement: Supplementary file 1 [file Table_1.DOCX]

Supplementary Figure 1 Distribution of SNP in chromosome


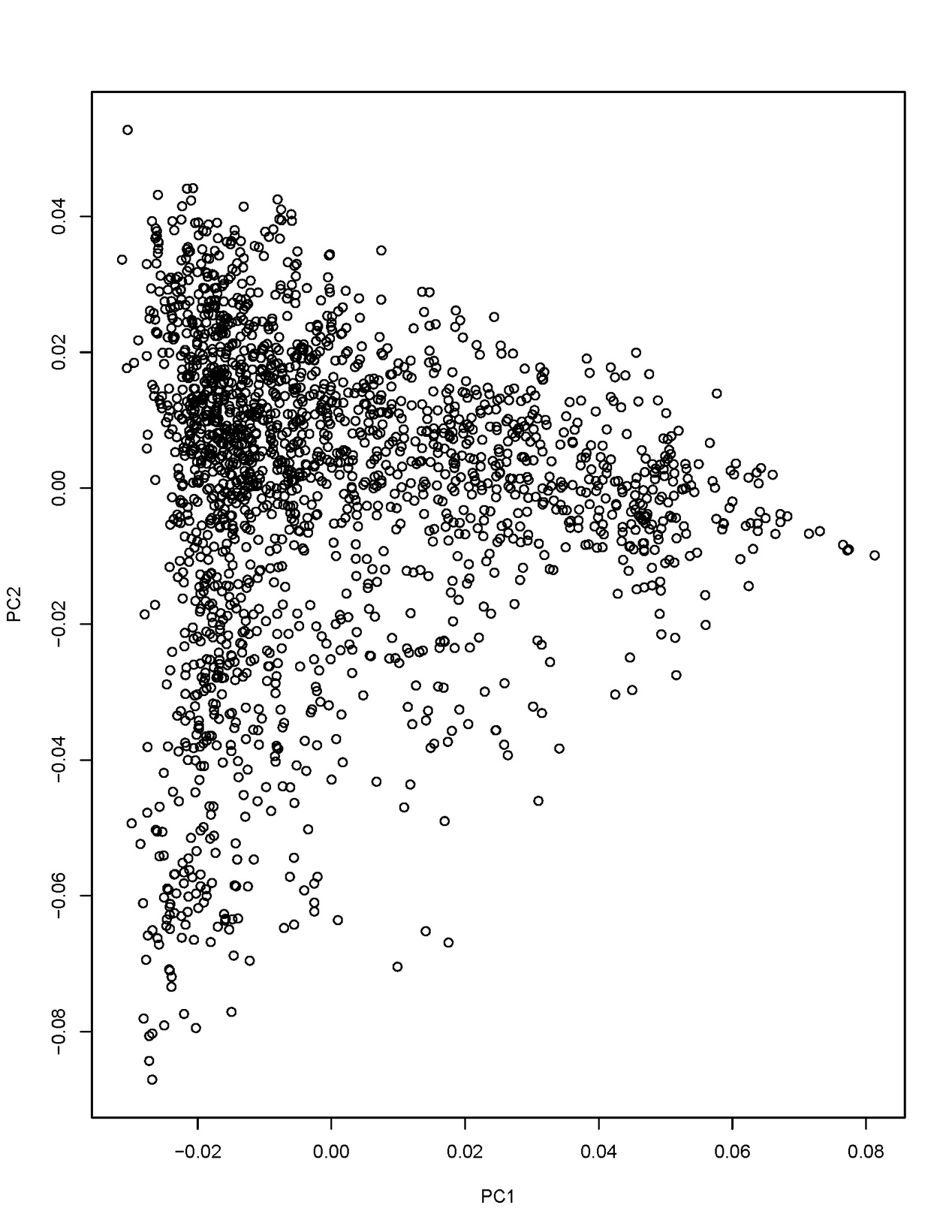


Supplementary Figure 2 Principal component analysis for IMCGs populations
